# Editorial: Harmonization of protocols investigating natural products: the role of chemical analysis from *in vitro* studies to clinical research

**DOI:** 10.3389/fphar.2024.1490109

**Published:** 2024-10-29

**Authors:** Mehmet Zeki Haznedaroglu, Luigi Menghini, Goksel Gokce, Alper Gokbulut

**Affiliations:** ^1^ Department of Pharmaceutical Botany, Faculty of Pharmacy, İzmir Katip Celebi University, Cigli, Turkiye; ^2^ Department of Pharmacy, University ‘G. d’Annunzio’ of Chieti-Pescara, Chieti, Italy; ^3^ Department of Pharmacology, Faculty of Pharmacy, Ege University, Bornova, Turkiye; ^4^ Faculty of Pharmacy, Ankara University, Ankara, Türkiye

**Keywords:** natural products, chemical analysis, ethnophamacology, meta-analysis, secondary metabolites, optimization, harmonization

The natural products have been the main source of bioactive chemicals throughout history. The term could refer to economic, legal, and health-related domains, as well as to agricultural and food products; sometimes even raising concerns about illegal substances. The expanding understanding of natural products now encompasses GMO-free requirements, along with concerns about toxicity and perishability (Stanziani, 2008).

Despite these complexities, the sales rates worldwide continue to grow, besides in the number of scientific articles. The natural and organic products market, including food and beverages, dietary supplements, had tripled in size since 2007, from $97 billion to over $300 billion (Nutraceuticalsworld, 2024). In the U.S. sales are projected to surpass $300B by 2024 and estimated as $400B by 2030 (Newhope, 2022). According to the Statista report, the global nutraceutical market was worth approximately $383B in 2017 (Statistics, 2021). Germany had the largest market in Europe with about €15.3B, followed by France with sales reaching almost €12.1B (Statista, 2024).

One of the early classifications as poisonous plants was named following the observation of potential of the toxicity of natural products. While those plants were used with caution since ancient times, nowadays some isolated metabolites also from toxic plants are thought to be natural and harmless that leads them to being used recklessly. Currently these bioactive compounds have turned into natural, harmless agents to be used in the treatment besides all other usages, without considering their potential involvement in the toxicity of origin plant. A huge amount of scientifical data is nowadays available on natural products but summarize in a unique interpretation for applicative potentialities are still a hard challenge due to different research approaches that includes different raw material, chemical characterization and bio-pharmaco-toxicological tests also with critical interpretation of effects related to single compound alone or in the context of complex matrix. Therefore, the standardization harmonization and regulation studies including interaction and adverse reactions are getting more important day by day.

This project aimed to collect high-quality review and original research articles to assess the analysis and harmonized procedures of plant extracts from the *in vitro* research to clinical trials. The topic gathered five manuscripts including three original and two review articles with an attraction of more than 18,000 views so far.

Pharmacologically active and novel secondary metabolites of four endophytic fungal community had been evaluated by Taritla et al. in an original article. *Sargassum muticum*, a marine brown alga, in the marine ecosystem was investigated for potential cyctotoxic and apoptosis induction. MTT assay on HeLa cells was performed on *Aspergillus* sp., *Nigrospora sphaerica, Talaromyces purpureogenus*, and *Talaromyces stipitatus*; further studies were performed for optimization such as, physicochemical parameters, growth curve, culture media, and organic solvents, and analysis was performed with GC-MS. The ethyl acetate extract of *Aspergillus* sp. led to apoptosis supported by ROS production, MMP depolarization, phosphatidylserine (PS) externalization, and activation of the caspase pathway.

A commonly used medicinal herb in Asian countries, Sulfur Angelicae Dahuricae Radix (Baizhi) had been studied to develop a practical protocol combining metabolomics, pharmacology, cytotoxicity and evaluate the influence of sulfur-fumigation on the quality for it. Isolation and purification analysis were performed with UPLC-QTOF-MS-Guided Marker. Following sulfur fumigation of Baizhi its chemical composition leaded to transformation of the coumarins into dihydrocoumarin sulfonic acids, and conversion of (4R, 12S)-3,4-dihydrooxypeucedanin hydrate-4-sulfonic acid into oxypeucedanin hydrate. The study showed that sulfur fumigation could reduce toxicity. Although chemical composition changed following the procedure there was no difference in the anti-inflammatory activity of Baizhi was studied by Ping Deng et al.

Huang et al. demonstrated in their meta-analysis that rhubarb has therapeutical benefits in chronic renal failure. The randomized and semi randomized controlled trials of rhubarb reached a total of 2,786 patients in 34 literatures, including 1,474 cases in the treatment group and 1,312 cases in the control group. In comparison with the control group, rhubarb could significantly reduce serum creatinine, blood urea nitrogen and uric acid, while increasing creatinine clearance rate, besides improving the total effective rate of symptoms and signs. In the conclusion the necessity of further studies with high-quality literature to evaluate its efficacy and safety was remarked.

The *Zingiber* and *Alpinia* species (Zingiberaceae), a popular supplement that had been used as food, spice is also commonly known with usages in ethnomedicine had been highlighted with the immunomodulatory properties, the bioactive metabolites. Bioactive secondary metabolites, of selected *Zingiber* and *Alpinia* species were included in the manuscript. Among many constituents particularly gingerols, and zerumbone have been indicated with the immunomodulating potential. The requirement of more mechanistic studies on the bioactive secondary metabolites had been emphasized by Jantan et al.

Kaczorova et al. focused on the extractive capability of various pharmaceutical ointment bases for phytocannabinoids of different *Cannabis sativa* L. chemotypes. Analysis were performed by uHPLC coupled to a UV detector. The stability during storage was investigated resulting with the olivae oleum and Synderman (SydoFarm^®^) extracts showing the best stability of all tested while the cream bases were the least stable and problematic for extract preparation. Additionally, a protocol of the preparation of cannabis-based galenic formulations for dermatological applications was proposed. The study also highlights the potential of phytocannabinoids for many skin disorders and its not being accepted in Czech regulations.

This Research Topic reflects the interest in natural products; potential solutions and projections of it. Recent review proposed natural products for combating cancer multidrug resistance (Chen et al., 2024). New technologies in the area help prospection of natural products (de Medeiros et al., 2023). Additionally, plant metabolomics, was defined as a rapidly advancing field for comprehensively exploring the small molecules (Kumar and Jaitak, 2024). Further aspects could be a database for the negative studies aiming activity and screening including the comprehensive material and method that would help sustainability and avoid duplication of unsuccessful efforts. More studies on the effects of the secondary metabolites with mechanism of action, preclinical pharmacokinetics, pharmacodynamics, bioavailability, toxicity studies and well conducted clinical trials and meta-analysis will always be required.
